# Piglet cardiopulmonary bypass induces intestinal dysbiosis and barrier dysfunction associated with systemic inflammation

**DOI:** 10.1242/dmm.049742

**Published:** 2023-01-12

**Authors:** Jeffrey D. Salomon, Haowen Qiu, Dan Feng, Jacob Owens, Ludmila Khailova, Suzanne Osorio Lujan, John Iguidbashian, Yashpal S. Chhonker, Daryl J. Murry, Jean-Jack Riethoven, Merry L. Lindsey, Amar B. Singh, Jesse A. Davidson

**Affiliations:** ^1^ Department of Pediatrics, University of Nebraska Medical Center, Omaha, NE 68102, USA; ^2^ Department of Cellular & Integrative Physiology, University of Nebraska Medical Center, Omaha, NE 68102, USA; ^3^ Center for Biotechnology, University of Nebraska Lincoln, Lincoln, NE 68588, USA; ^4^ Department of Hematology/Oncology, University of Nebraska Medical Center, Omaha, NE 68102, USA; ^5^ Department of Pediatrics, University of Colorado, Aurora, CO 80045, USA; ^6^ Department of Pharmacy Practice, University of Nebraska Medical Center College of Pharmacy, Omaha, NE 68102, USA; ^7^ School of Graduate Studies and Research, Meharry Medical College, Nashville, TN 37208, USA; ^8^ Research Service, Nebraska-Western Iowa Health Care System, Omaha, NE 68105, USA; ^9^ Department of Biochemistry & Molecular Biology, University of Nebraska Medical Center, Omaha, NE 68102, USA

**Keywords:** Congenital heart disease, Microbiome, Inflammation, Barrier dysfunction, Short-chain fatty acid

## Abstract

The intestinal microbiome is essential to human health and homeostasis, and is implicated in the pathophysiology of disease, including congenital heart disease and cardiac surgery. Improving the microbiome and reducing inflammatory metabolites may reduce systemic inflammation following cardiac surgery with cardiopulmonary bypass (CPB) to expedite recovery post-operatively. Limited research exists in this area and identifying animal models that can replicate changes in the human intestinal microbiome after CPB is necessary. We used a piglet model of CPB with two groups, CPB (*n*=5) and a control group with mechanical ventilation (*n*=7), to evaluate changes to the microbiome, intestinal barrier dysfunction and intestinal metabolites with inflammation after CPB. We identified significant changes to the microbiome, barrier dysfunction, intestinal short-chain fatty acids and eicosanoids, and elevated cytokines in the CPB/deep hypothermic circulatory arrest group compared to the control group at just 4 h after intervention. This piglet model of CPB replicates known human changes to intestinal flora and metabolite profiles, and can be used to evaluate gut interventions aimed at reducing downstream inflammation after cardiac surgery with CPB.

## Teaser

**Summary:** Using a piglet model of cardiopulmonary bypass, we demonstrate significant changes in the microbiome, a key regulator of health and homeostasis, and evidence of intestinal barrier dysfunction following cardiopulmonary bypass associated with systemic inflammation.

## INTRODUCTION

Congenital heart disease affects nearly 40,000 infants in the USA annually and occurs in approximately 1% of all live births worldwide (https://www.cdc.gov/ncbddd/heartdefects/data.html; Wu et al., 2020). Many congenital heart defects requiring surgical repair also require cardiopulmonary bypass (CPB). Additionally, the use of deep hypothermic circulatory arrest (DHCA) or selective cerebral perfusion might be used for organ protection during complex surgical repairs involving the major blood vessels leading to the brain and body (Conolly et al., 2010; Tintoiu et al., 2018). CPB is known to induce inflammation (Halter et al., 2005), and the inflammatory response following CPB with cardiac surgery is a significant cause of morbidity and mortality and can induce low cardiac output syndrome (LCOS). Roughly 20-25% of all pediatric patients experience some degree of LCOS, and around 50% of neonates develop LCOS (Chandler and Kirsch, 2016; Wessel, 2001; Cremer et al., 1996; Hammer et al., 2001; Sinclair et al., 1995). Within this population, those that develop LCOS have a higher mortality, increased intensive care unit (ICU) length of stay, and longer duration of mechanical ventilation (Du et al., 2020). Uncovering participating factors contributing to the inflammatory process through regulation of the intestinal microbiome, intestinal epithelial barrier dysfunction (EBD) and resultant metabolites might contribute to our understanding of systemic inflammation (Salomon et al., 2021; Typpo et al., 2015).

The microbiome plays a critical role in maintaining human health and homeostasis and acts as a driver of the pathology, including heart disease (Forkosh and Ilan, 2019; Jia et al., 2019; Kitai et al., 2016; Wang et al., 2018). Alterations to the general makeup of the microbiome, such as reductions in α-diversity and β-diversity, can influence how the microbial system interacts with the host (Reese and Dunn, 2018). Specific bacterial shifts, including reductions in short-chain fatty acid (SCFA)-producing organisms and increases in pro-inflammatory bacteria, which can produce inflammatory eicosanoids, can alter inflammatory metabolite production, resulting in exacerbation of inflammatory processes (Bull and Plummer, 2014; Cabrera-Perez et al., 2017; Wang et al., 2018; Ferrer and Moreno, 2010; Salomon et al., 2021; Sheppe and Edelmann, 2021). SCFAs and eicosanoids interact with the intestinal microbiome to regulate barrier integrity and inflammation (Parada Venegas et al., 2019; van der Hee and Wells, 2021; Ohira et al., 2017). SCFAs are also cardioprotective and reduced levels of these metabolites have been associated with inflammation, heart failure and myocardial ischemia (Tang et al., 2019; Chen et al., 2020; Ohira et al., 2017; Pakhomov and Baugh, 2021). Although SCFAs help maintain the intestinal barrier, CPB is known to induce intestinal EBD, resulting in gut permeability (Salomon et al., 2021; Typpo et al., 2015; Al-Sadi et al., 2009; Ahmad et al., 2017; Yonker et al., 2021). Markers of EBD include claudin-2 (CLDN2) and claudin-3 (CLDN3), which are intercellular tight-junction proteins serving as gatekeepers for molecules, and fatty acid-binding protein 2 (FABP2), a lipid transporter regulating cell homeostasis (Luettig et al., 2015; Milatz et al., 2010; Lau et al., 2016). When these markers are found in the circulation, it is indicative of EBD. The presence of EBD and intestinal permeability is considered a mediator for dysbiosis and for metabolites to leak out of the intestinal tract and exacerbate systemic inflammation (Salomon et al., 2021).

Many animal models have been useful in evaluating CPB and a variety of cardiovascular, kidney, respiratory and neurologic outcomes (Davidson et al., 2019; Grocott et al., 1999; Hubert et al., 2003; Jungwirth and De Lange, 2010; Madrahimov et al., 2018). No animal models of CPB, however, have been utilized to evaluate changes to the microbiome, intestinal barrier dysfunction or intestinal eicosanoids. As the importance of the microbiome grows, animal models are crucial to evaluate the microbiome and factors contributing to post-surgical inflammation to identify potential therapeutic interventions. In this article, we used a model of piglet CPB/DHCA to evaluate the microbiome, intestinal EBD, SCFAs and eicosanoids. We hypothesized that the CPB/DHCA group would experience microbial and metabolite derangements along with EBD and systemic inflammation compared to controls.

## RESULTS

A total of 12 piglets were used in this study, five in the CPB/DHCA group and seven in the control group receiving mechanical ventilation only. Table 1 shows the mean doses of medications and vasoactive inotrope score (VIS) for the CPB/DHCA group piglets indicating the level of support required during the period off bypass. Piglet #1 in the CPB/DHCA group was noted to have higher hemodynamic support requirements during the 4 h between separation from CPB and euthanasia, with the VIS continuing to escalate during the supportive period, and this correlated with other variables mentioned in subsequent sections. Individual VIS and vasoactive medication doses for each piglet are summarized in Table S1. The samples used in the analysis were collected after initiation of anesthesia and at sacrifice for all stool and serum.

**Table 1.**
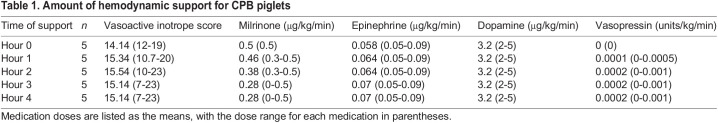
Amount of hemodynamic support for CPB piglets

### Microbiome

**Fig. 1.**
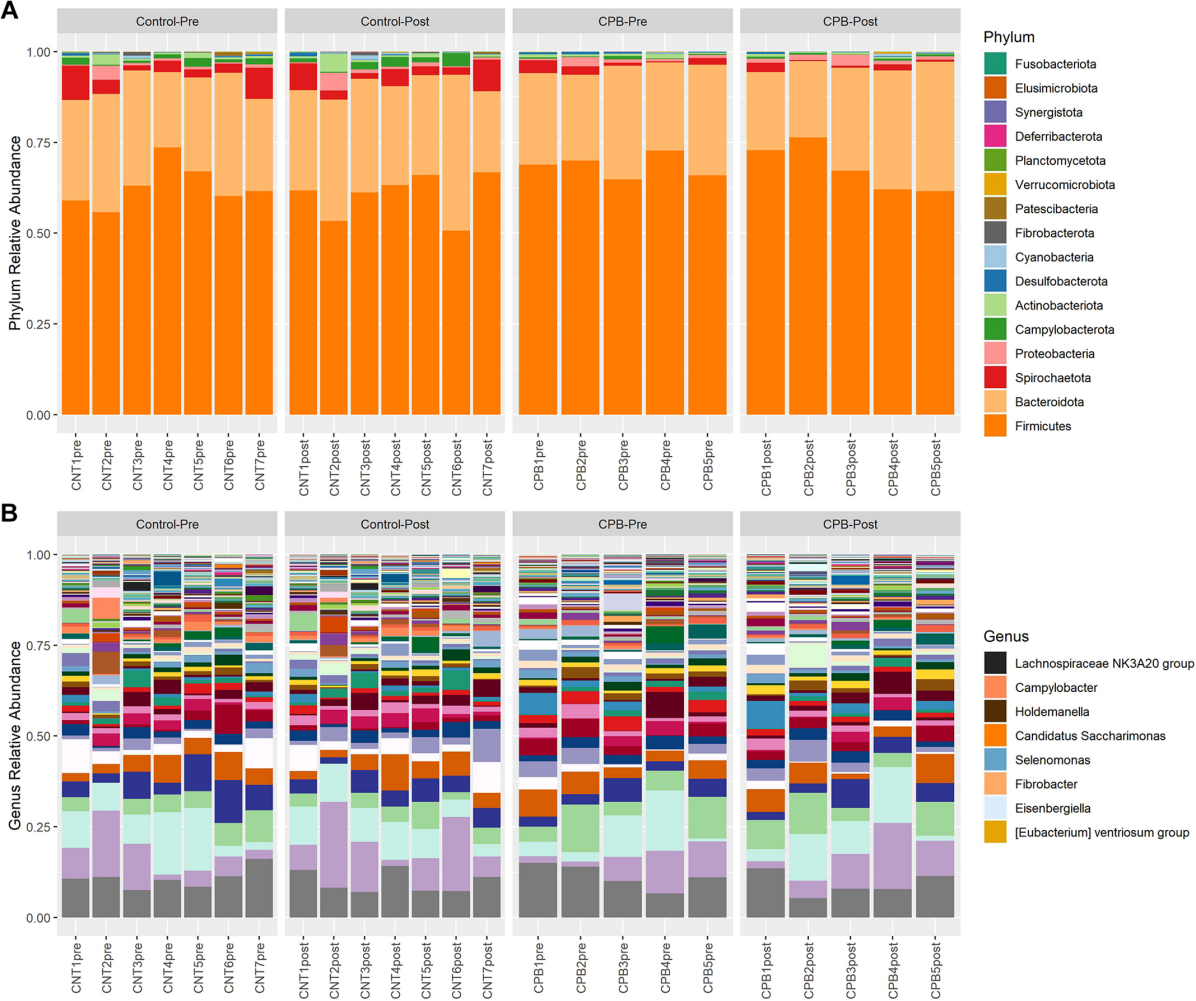
**Relative bacterial abundance between CPB/DHCA group and controls.** (A) Bacteria at the phylum level in each sample pre- and post-surgery for the control group and the CPB/DHCA group. There is a slightly larger increase in the amounts of Proteobacteria in the CPB/DHCA group pre-operative to post-operative samples compared to the control group. (B) Bacteria at the genus level. The legend identifies SCFA-producing organisms, which are reduced in the CPB/DHCA group post-operative samples compared to the control group post-operative samples. CPB, cardiopulmonary bypass; SCFA, short-chain fatty acid.

**Fig. 2.**
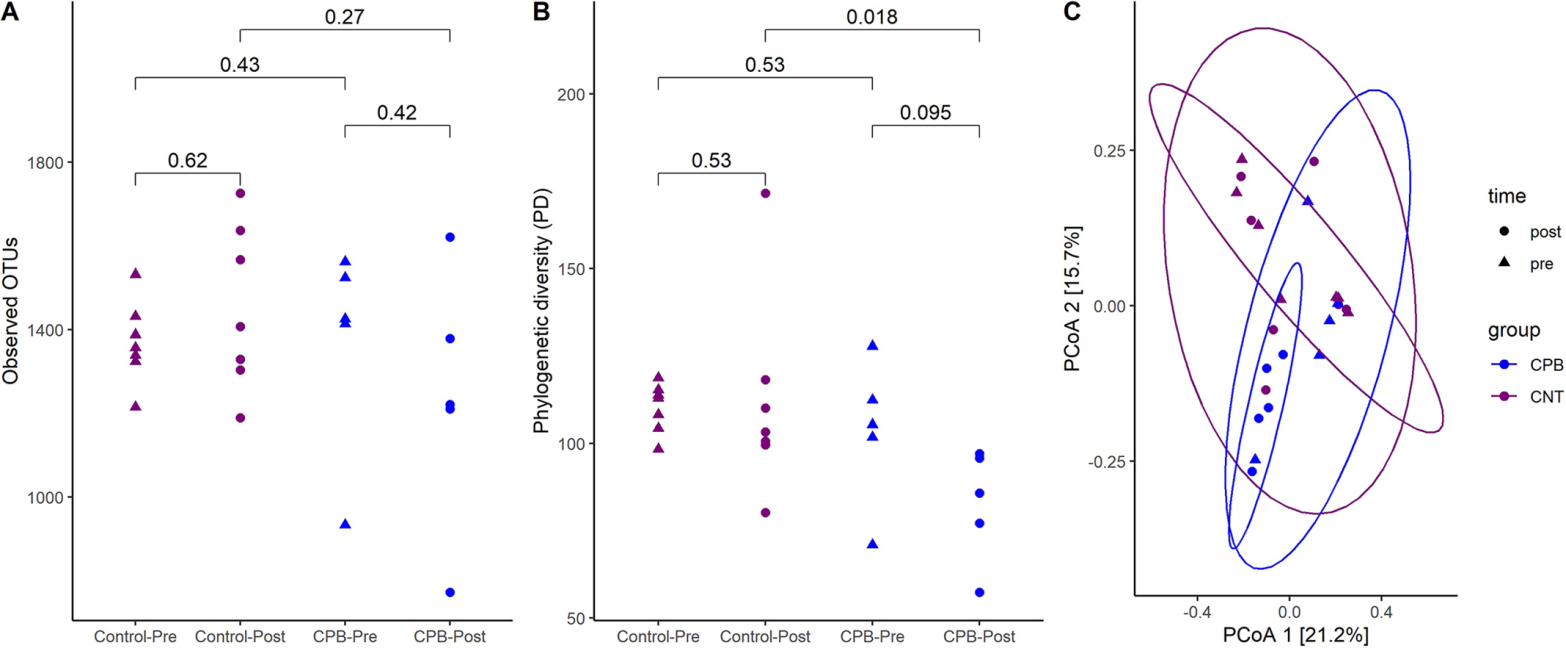
**α- and β-diversity plots in CPB/DHCA group and controls.** (A) Observed operational taxonomic units (OTUs) in the CPB/DHCA group compared to the control group. There were no statistically significant differences in the total number of bacteria present between the two groups. (B) Phylogenetic diversity between the CPB/DHCA group and the control group. There was a significant decrease in phylogenetic diversity in the CPB post-operative samples compared to the control post-operative samples. (C) β-diversity via UniFrac distance matrix. There was a statistically significant difference in the β-diversity in the CPB group compared to the control group. The numbers indicate *P*-values using unpaired Wilcoxon rank sum test. PCoA, principal coordinates analysis.

Sequences of 16S rRNA amplicon libraries generated using fecal microbial DNA resulted in a total of 12.6 million reads. The relative abundance plot shows the taxonomic distribution in each group at the phylum and genus levels (Fig. 1). Although the Firmicutes and Bacteroidota phyla dominated both groups, the CPB/DHCA group trended towards a reduced number of different species in the post-operative samples compared to those in the pre-operative samples. The bacterial richness, indicated by the number of distinct operational taxonomic units (OTUs) identified in each sample, was not significantly different between the two groups (Fig. 2A). Phylogenic diversity showed a significant difference between the control and CPB/DHCA group in the post-operative time point (*P*=0.018, Fig. 2B). There was a trend towards significant reduction in α-diversity between the pre- and post-operative samples in the CPB/DHCA group (*P*=0.095).

Overall β-diversity (i.e. inter-subject differences in community composition) was visualized using principal coordinates analysis (PCoA) and evaluated statistically with permutational multivariate ANOVA (PERMANOVA). There were significant differences in β-diversity between groups using all four distance matrices (Bray–Curtis, *P*=0.017; Jaccard, *P*=0.003; UniFrac, *P*=0.018; and weighted UniFrac, *P*=0.017). β-diversity using UniFrac distance matrix also showed a trend toward significance after first blocking by time point (Fig. 2C), suggesting that the group undergoing CPB/DHCA is the dominant factor driving microbiome community dissimilarities.

To identify specific taxonomic variations associated with group (CPB/DHCA versus control), differential abundance analyses were performed that identified multiple groups of organisms at the genus, family and phylum level with significant abundance differences between the CPB/DHCA group and the controls. At the genus level, SCFA-producing organisms, such as *Fibrobacter*, *Eisenbergiella*, *Campylobacter*, *Lachnispiraceae* NK3A20 group and the *Eubacterium* genera, among others, were reduced in the CPB/DHCA group compared to the controls (Fig. S1A) (Sun et al., 2021; Li et al., 2020). At the family level, similar groups of SCFA-producing organisms, such as Spirochaetaceae, Selenomonadaceae, Christensenellaceae and Fibrobacteraceae, were reduced in the CPB/DHCA group compared to the controls (Fig. S1B) (Peterson et al., 2022; Li et al., 2020; Liu et al., 2019; Mukherjee et al., 2020; Walker et al., 2005; Van den Abbeele et al., 2022).

**Fig. 3.**
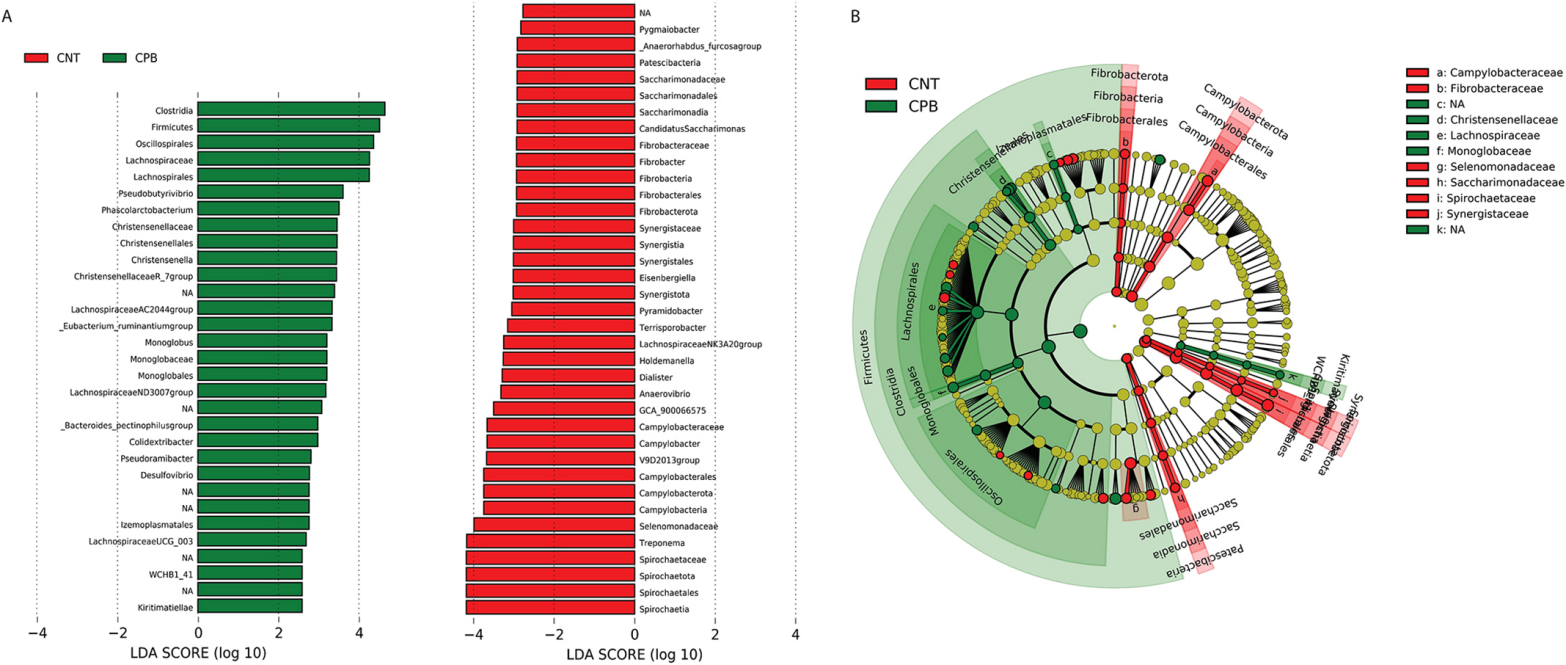
**LEfSE plot and cladogram of bacterial associations in CPB/DHCA group and controls.** (A) LEfSE plot providing organisms associated with either the CPB/DHCA group (green) or the control group (CNT, red). The logarithmic score details the strength of the association of each organism to a specific group. (B) Cladogram of the LEfSE analysis with organisms in the shaded green area associating more strongly with the CPB/DHCA group and organisms in the shaded red area associating with the control group. The microbial compositions were compared at different taxonomic levels. LDA, linear discriminate analysis.

Linear discriminant analysis (LDA) effect size (LEfSE) was performed to identify microbial biomarkers at different classification levels between the two groups (LDA score>2.0). This is used to determine the features most likely to explain differences between groups by coupling standard tests for statistical significance with additional tests encoding biological consistency and effect (Segata et al., 2011). LEfSE revealed multiple genera predominantly associated with either the CPB/DHCA group or the control group (Fig. 3A). A cladogram was developed for taxonomic representation of biologically consistent differences in the CPB/DHCA group and the control group (Fig. 3B). Broadly, several families of organisms were noted to be associated with the CPB/DHCA group in both the LEfSE plot and cladogram, including Lachnospiraceae, Christensenellaceae, Monoglobaceae and Peptococcaceae.

### EBD markers, inflammatory cytokines and short-chain fatty acids

**Fig. 4.**
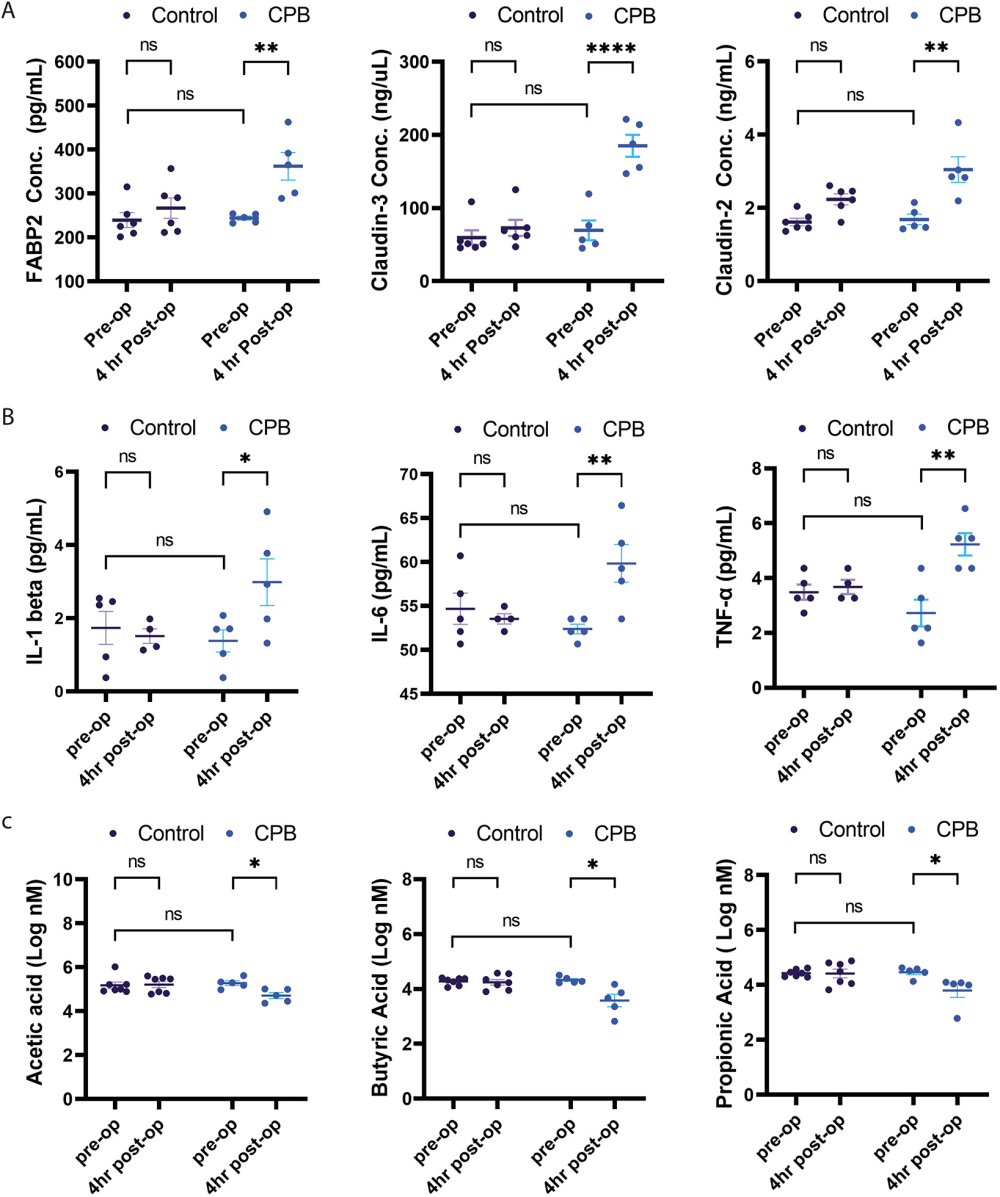
**Changes in markers of EBD, cytokines and SCFAs between the CPB/DHCA group and controls.** (A) A significant increase was seen in FABP2, claudin-2 and claudin-3 levels between the pre-operative and post-operative samples in the CPB group compared to controls. (B) A significant increase was seen in IL-1β, IL-6 and TNF-α levels between the pre-operative and post-operative samples in the CPB/DHCA group compared to controls. (C) A significant reduction was seen in acetic acid, butyric acid and propionic acid levels between the pre-operative and post-operative samples in the CPB group compared to controls. Two-way ANOVA with Holm–Sidak's multiple comparisons test was performed. FABP2, fatty acid-binding protein 2; IL, interleukin; TNF, tumor necrosis factor. ns, not significant; *P<0.05; **P<0.01; ****P<0.0001.

Serum markers of intestinal barrier dysfunction were obtained from all animals. Fig. 4A demonstrates the levels of FABP2, claudin-2 and claudin-3 in the arterial serum samples in the CPB/DHCA group versus controls. There were no significant differences for any of the EBD markers in the pre-operative samples in either group. A significant increase was identified in FABP2 (*P*<0.05), claudin-2 (*P*<0.0001), and claudin-3 (*P*<0.01) between the pre-operative and post-operative samples in the CPB/DHCA group but not in the controls. Interestingly, the piglet requiring the highest amount of cardiovascular support, piglet #1 in the CPB/DHCA group, was also noted to have the greatest increase in claudin-2 (56 to 214 ng/μl) and claudin-3 (1.43 to 4.33 ng/ml).

Serum inflammatory cytokines were measured in pre-operative and post-operative samples for both CPB/DHCA and control groups. Fig. 4B demonstrates the levels of IL-1β, IL-6, and TNF-α (encoded by *IL1B*, *IL6* and *TNF*, respectively). There were no significant differences for any of the cytokines in the pre-operative samples between groups. In the CBP/DHCA group, IL-1β (*P*<0.05), IL-6 (*P*<0.01) and TNF-α (*P*<0.01) were all increased in the post-operative samples compared to the pre-operative samples. Specifically, piglet #1 in the CPB/DHCA group, which was noted to have both the highest total VIS and continually rising VIS during the supportive period, was also noted to have the largest increase in both IL-1β (0.28 to 4.91 pg/ml) and IL-6 (50.67 to 66.45 pg/ml) between the pre-operative and post-operative samples. No cytokines in the control group had a significant increase between the pre-operative and post-operative samples.

Several SCFAs were significantly reduced in the 4-h post-operative stool samples compared to the pre-operative samples in the CPB/DHCA group, with no significant change noted for the control group. The levels of three prominent SCFAs, acetic acid (*P*=0.024), butyric acid (*P*=0.011) and propionic acid (*P*=0.018) are shown in Fig. 4C. Additional SCFAs, such as valeric acid, isovaleric acid and 2-methyl butyric acid, were also noted to have *P*<0.05.

### Intestinal eicosanoids

**Fig. 5.**
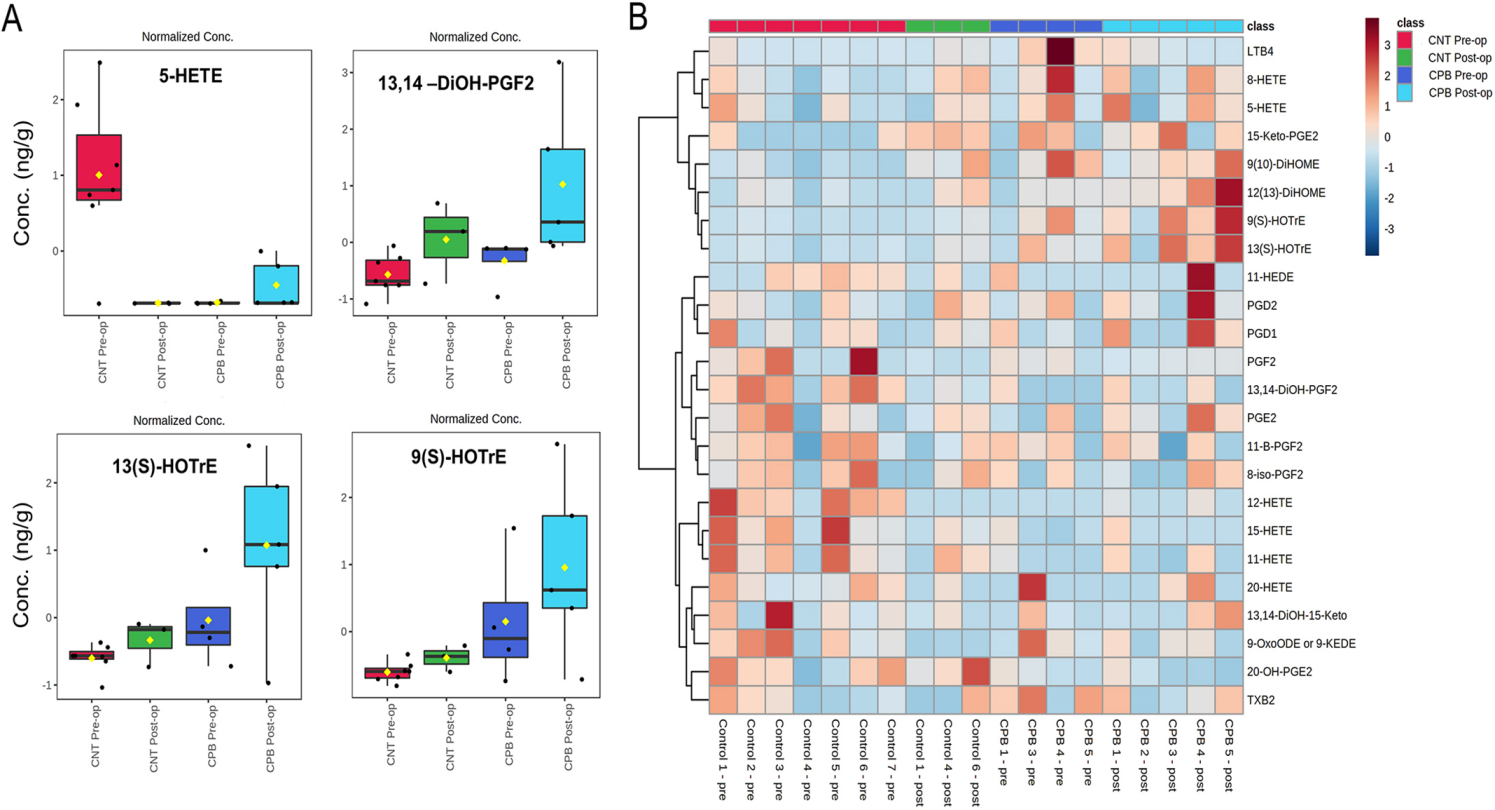
**Changes to stool eicosanoids between CPB/DHCA group and controls.** (A) A selection of eicosanoids with variation between the CPB/DHCA group and the control (CNT) group. Boxes indicate the 25-75th percentiles, whiskers show 1.5 times the interquartile range, the central line marks the median, and the mean is indicated by the yellow diamond. (B) A heatmap plot of the association of various eicosanoids with both pre-operative and post-operative samples of the CPB/DHCA group and controls. The Mann–Whitney U test was used to assess differences between the two groups. Conc, concentration; HETE, Hydroxyeicosatetraenoic acid; DiOH-PGF2, dihydro-prostaglandin 2; HOTrE, hydroxyoctadecatrienoic acid.

A panel of 66 eicosanoids was evaluated (Chhonker et al., 2021) for stool samples, with many falling below the detectable range. Those eicosanoids that were below a detectable range of 0.01 ng/ml were removed from the analysis. We identified changes in multiple stool eicosanoids between the CPB pre-operative and CPB post-operative samples, such as 12-hydroxyeicosatetraenoic acid (12-HETE), 9S-hydroxy-10E,12Z,15Z-octadecatrienoic acid [9(S)-HOTrE], 13S-hydroxy-9Z,11E,15Z-octadecatrienoic acid [13(S)-HOTrE] and 13-14-dihydro-15-keto-prostaglandin F2 (13,14-diOH-PGF2) (Fig. 5A). A heat map was created to reflect changes of different eicosanoids between groups and time points (Fig. 5B). There were sample limitations for analysis with a different number of animals analyzed between each group owing to the amount of stool available. The control group had *n*=7 in the pre-operative samples and *n*=3 in the post-operative samples. The CPB/DHCA group had *n*=4 in the pre-operative samples and *n*=5 in the post-operative samples. We noted shifts in the eicosanoids present between the two groups, indicating unique eicosanoid profiling within the CPB/DHCA group. These include upregulation of prostaglandins D1 (PGD1) and D2 (PGD2), 9(S)-HOTrE and 13(S)-HOTrE, and a downregulation of 12-HETE, 15-hydroxyeicosatetraenoic acid (15-HETE) and prostaglandin E2 (PGE2) in CPB versus controls. Partial least squares discriminate analysis (PLS-DA) was performed and depicts the shift in stool eicosanoids between the two groups at each time point (Fig. S2A), and VIP scoring measured the importance of each variable in the PLS-DA model (Fig. S2B).

### Canonical correlation analysis for microbiome and mediation analysis

**Fig. 6.**
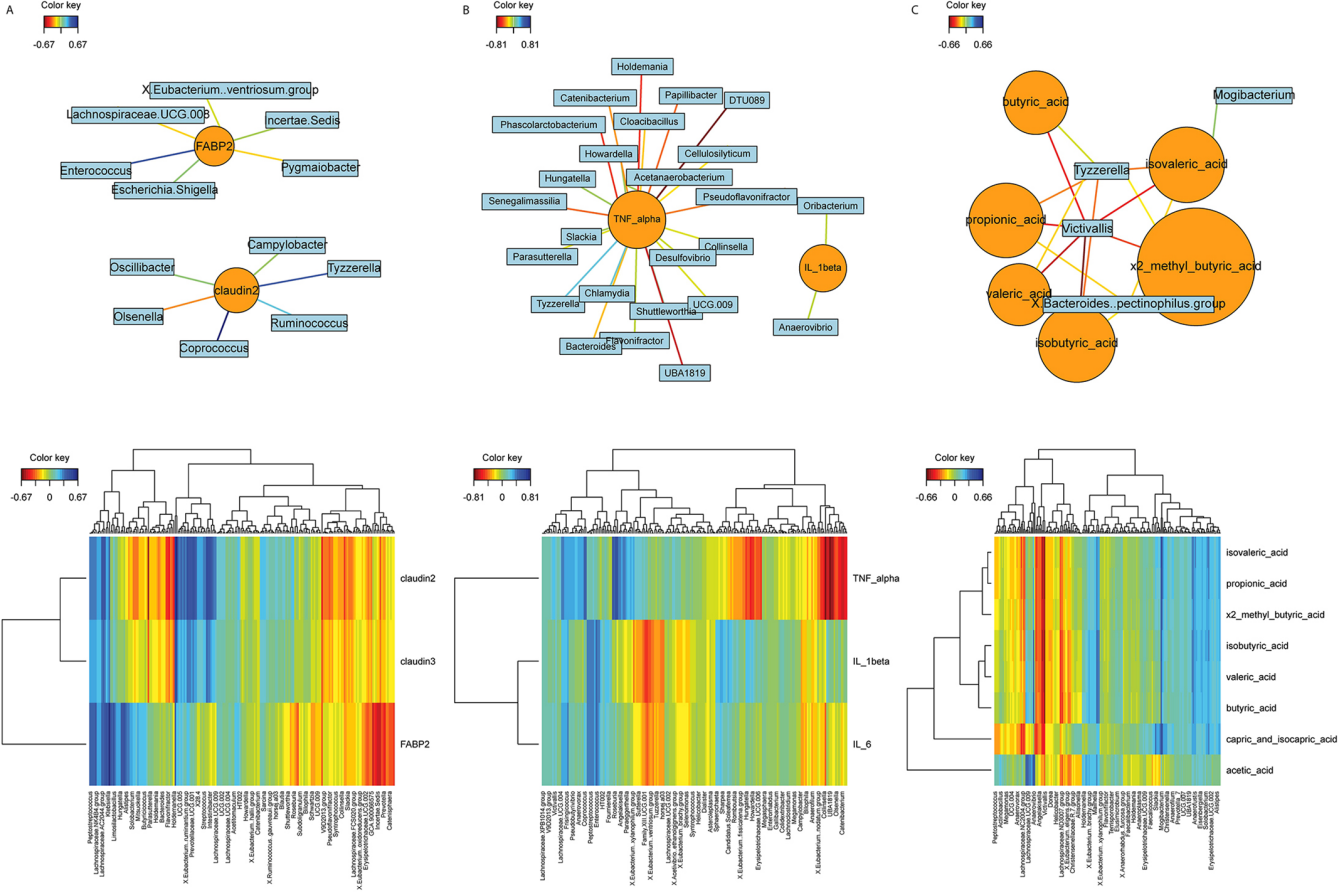
**Canonical correlation analysis of the microbiome with EBD, cytokines and SCFA.** (A) Network map (top) and heatmap (bottom) of the markers of EBD and associated organisms. (B) Network map (top) and heatmap (bottom) of inflammatory cytokines and associated organisms. (C) Network map (top) and heatmap (bottom) of SCFAs and associated organisms.

Canonical correlation analysis was performed to evaluate the correlation between the microbiome and other sets of measured biomarkers. Network and heatmap analysis showed the strength of association between the microbiome and these biomarkers. The markers for EBD (Fig. 6A), specifically FABP2, were positively associated with pro-inflammatory organisms, such as *Klebsiella*, *Escherichia* and *Enterococcus*, and negatively associated with the SCFA-producing organisms *Roseburia*, Lachnospiraceae UCG.008, and *Eubacterium*. Claudin-2 was noted to have negative association with *Holdemania*, an SCFA-producing organism, as well as increases in *Klebsiella* and *Peptostreptococcus*, known to induce intestinal inflammation (Atarashi et al., 2017). The cytokine network and heatmap (Fig. 6B) demonstrated that many organisms were negatively associated with TNF-α, but some had a positive association with IL-1β and IL-6, such as *Hungatella*, *Howardella* and *Romboutsia*. Conversely, *Roseburia*, *Fournierella* and *Angelakisella* were noted to have a strongly negative association with TNF-α and a mildly positive or neutral association with IL-1β and IL-6. Specifically looking at SCFAs (Fig. 6C), *Victivallis* had significant negative association with all SCFAs in both the network and heatmap.

**Fig. 7.**
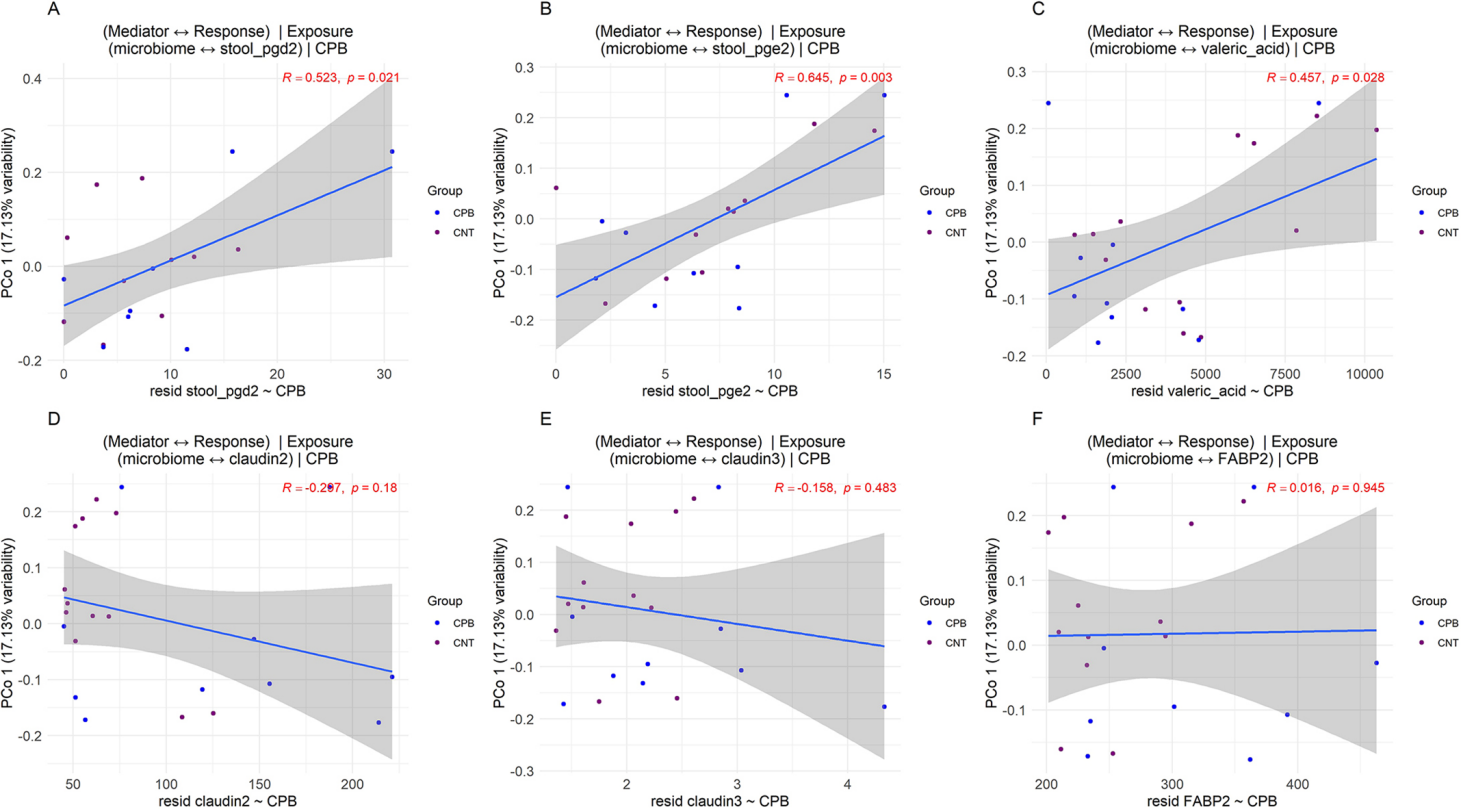
**Mediation analysis of the microbiome on changes to EBD, cytokines, SCFAs and eicosanoids.** (A) The three outcomes, PGD2, PGE2 and valeric acid, to be mediated by changes in the microbiome. Exposure is CPB, the mediator is the microbiome, and the outcomes are listed above. (B) The three markers of EBD, FABP2, claudin-2 and claudin-3, depicting no statistically significant mediation effect of the microbiome on the changes in EBD. CNT, control; FABP2, fatty acid-binding protein 2. Blue lines indicate the association between the microbiome and individual biomarkers using principal component axis 1, and shaded gray areas represent the 95% confidence intervals.

Mediation analysis was performed to further understand the role the microbiome played as a mediator for outcomes such as other measured biomarkers, using CPB/DHCA as the exposure. We identified two eicosanoids, PGD2 and PGE2, along with valeric acid to be significantly mediated by the microbiome, given CPB as exposure (Fig. 7A). As expected, the microbiome was not a significant mediator for intestinal EBD (Fig. 7B), which corroborates the theory that CPB directly induces intestinal barrier dysfunction, thereby creating the intestinal permeability for the microbiome and intestinal metabolites to leak out of the gut and signal systemic inflammation.

## DISCUSSION

This is the first study to use a piglet model of CPB with DHCA to evaluate the intestinal microbiome, EBD and the intestinal metabolite profile. Despite the short window between the pre-intervention samples and the post-intervention samples in the two groups, there were significant changes noted in multiple areas of interest, including the microbiome, intestinal EBD, cytokines, SCFAs and eicosanoids. Within just a few hours, differences in α-diversity and β-diversity of the intestinal microbiome were already present. Coinciding with the microbial changes, the stool metabolite profile also demonstrated important shifts with a reduction of SCFAs as well as evidence of significant barrier dysfunction. Associated with these intestinal flora and metabolite changes, there was also evidence of systemic inflammation as measured by circulating pro-inflammatory cytokines. Taken together, these results show CPB is associated with microbial and metabolite derangements in the gut along with barrier dysfunction and systemic inflammation. These changes occurred in the hyperacute period following CPB, which supports the possibility of a causal role in these events rather than the result of complex post-operative management occurring in patients after cardiac surgery.

Although it is still common practice to utilize rodent models for studies involving the microbiome and disease, there are distinct advantages to utilizing a porcine model to evaluate changes to the intestinal microbiome. The pig intestinal microbiome shares more similarities to humans than rodents, especially in longitudinal colonization (Aluthge et al., 2020). Additionally, pigs are more genetically similar to humans, and pig epithelia and other tissue types share more developmental and anatomical features with humans than rodent models; this increases the translational capabilities of evaluating changes in microbiota and intestinal injury to human subjects (Hvistendahl and De Lange, 2012).

SCFA-producing organisms were decreased in the CPB/DHCA group. As the microbiota producing SCFAs decreased (*Selenomas*, *Holdemanella* and *Lachnospiraceae*) (Atasoglu et al., 1998; Romani-Perez et al., 2021; Zhang et al., 2019), the amount of SCFA identified in the intestinal tract also decreased. As SCFAs contribute to intestinal barrier integrity, influence inflammatory signaling, and are cardioprotective, reductions in SCFAs in the piglets following CPB with DHCA might modulate the amount of inflammation and cardiac depression noted after CPB. Additionally, the pro-inflammatory organisms in the phyla Psuedomonadota (previously named Proteobacteria), such as *Escherichia*, *Sutterella*, *Burkholderia*, *Succinivibrio* and *Actinobacillus*, and eicosanoids in the intestinal tract can upregulate inflammatory signaling (Zargar et al., 2015; Ganesan and Sajjan, 2011; Hiippala et al., 2016). This process may be facilitated through intestinal EBD, resulting in increased permeability and leak of these inflammatory mediators into the circulation. These mediators can increase activation of inflammatory pathways, such as NF-κB and HIF-1α (Palazon et al., 2014; Sun et al., 2019). Diet and the environment also play a role in the production of metabolites geared to increase NF-κB production and pro-inflammatory gene modification (Rao and Lokesh, 2017).

Our group previously demonstrated evidence of EBD following bypass (Salomon et al., 2021; Watson et al., 2020), along with Typpo et al. (2015). Although the process through which these proteins transition from the intestinal epithelium into systemic circulation remains unclear, it is well documented that increasing amounts of these proteins in the circulation correlates with barrier dysfunction and, by extension, increases intestinal permeability (Typpo et al., 2015; Lau et al., 2016; Ahmad et al., 2017; Luettig et al., 2015; Yonker et al., 2021). Here as well, we demonstrated evidence of intestinal EBD in the CPB/DHCA group based upon dysregulated levels of claudin-2, claudin-3 and FABP2. These perturbations of the intestinal barrier and intestinal permeability can allow leakage of bacteria, toxins and inflammatory metabolites into the deeper tissues, and eventually into the systemic circulation where they can continue activation of the inflammatory cascade. Typpo et al. (2015) also demonstrated a direct correlation of certain markers of EBD with fluid overload and the arteriovenous oxygen difference, which suggests a response to hypoxemia or poor cardiac output in children with congenital heart disease following CPB. This will be evaluated in future studies in which the piglets are supported for 5 days following CPB. The presence of EBD occurred concurrently with microbial changes, reduced SCFA, and increased circulating cytokines. Our canonical correlation analysis was able to detect a strong correlation between specific microbial changes and the presence of EBD in the CPB/DHCA group compared to controls.

It is well established that SCFAs are involved in mucosal and systemic inflammatory regulation, play a role in maintaining the intestinal barrier, and are cardioprotective (Chen et al., 2020; Li et al., 2020; Parada Venegas et al., 2019; Peterson et al., 2022; Tang et al., 2019; van der Hee and Wells, 2021). Reductions in SCFAs following CPB might result in reduced ability to downregulate inflammatory signaling. Targeting mechanisms to increase the production and amount of SCFAs prior to cardiac surgery with CPB might result in reduced post-operative inflammation. Additionally, with the multitude of other bacterial signaling through the gut–brain axis, gut–lung axis and gut–heart axis, an improved pre-operative microbiome and metabolite profile might reduce the inflammatory cascade in the post-operative state (Schmitt et al., 2019; Lau et al., 2021). Furthermore, a healthier pre-operative microbiome might result in improved nutritional status both pre-operatively and nutrient utilization in the post-operative period (Krajmalnik-Brown et al., 2012; Xie et al., 2021). Both components might result in faster wound healing and recovery. Further studies to examine interventions aimed at modulating the intestinal microbiome and assessing downstream inflammation and outcomes need to be performed.

We noted significant increases in three inflammatory cytokines, IL-1β, IL-6 and TNF-α, between the pre-operative and post-operative samples in the CPB/DHCA group compared to controls. A canonical correlation network map showed association with certain organisms in the CPB/DHCA group to cytokine changes. These included a negative correlation with many SCFA-producing organisms, such as *Holdemania*, *Howardella* and *Clostridiales* DTU089 (Chang and Yu, 2022; Li et al., 2019). Cytokines remain a crude but crucial component to evaluate measurable inflammation in both human and animal models. As the understanding and mechanistic links to inflammatory signaling are delineated (Jimenez-Aguilar et al., 2020; Robich et al., 2020), flow cytometry and RNA sequencing will be performed to better evaluate downstream systemic inflammatory changes.

The mediation analysis provided some interesting data evaluating the mediating effect of the microbiomes on downstream variable changes with CPB/DHCA as the exposure. PGE2 is known to induce acute inflammation through mast cell and Th-1 cell activation (Tsuge et al., 2019; Kawahara et al., 2015). PGD2, conversely, has been associated with anti-inflammatory signaling, but can promote or suppress inflammation depending on the inflammatory milieu (Joo and Sadikot, 2012; Murata and Maehara, 2016). Valeric acid has been associated with regulation of blood pressure mechanisms through angiotensin-converting enzyme inhibition, as well as protection from bacterial translocation (Takagaki and Nanjo, 2015; Peron et al., 2017). These metabolites may hold promising mechanistic links in how the microbiome can influence downstream inflammation and offers targets for future research to explore.

Although this study presents strong evidence of associations between CPB and the development of microbial and metabolite derangements along with barrier dysfunction and systemic inflammation, there are some limitations. This study had a small number of animals, which limits the ability to interpret change and the statistical significance of many markers. A study with more subjects may yield an improved understanding of these associations and identify additional targets for evaluation and intervention. Additionally, two interventions were utilized together in this study, CPB and DHCA. Many centers utilize selective cerebral perfusion instead of DHCA; one major center consistently using DHCA for Norwood procedures reported a median DHCA of 45 min with an intra-quartile range of 40-49 min (Sperotto et al., 2021). The duration of DHCA in this study was to overcome the healthy state of the piglets prior to intervention and attempt to induce a similar degree of inflammatory response and heart failure as seen with the most affected portion of our cardiac population. Future studies will evaluate the role of CPB without DHCA to explore a synergistic effect on the other variables. Hormones, especially estrogen, are known to influence the microbiome and inflammation (Baker et al., 2017; Monteiro et al., 2014). As this study only used female piglets, it is unclear whether sex hormones played a role in the measured variables. Interpretation of eicosanoids was made challenging by having a limited supply of serum to analyze all variables. Metabolomics were performed last, and some animals did not have enough serum to perform pre- and post-assessments. This is reflected in the box plots and heatmap in Fig. 5 and PLS-DA score plot in Fig. S2. Additionally, there was a brief period of time between the sample collections. It is unclear whether these changes are brief or persist for an extended time during the post-operative period. Human data would support evidence of EBD for at least 48-72 h and more pro-inflammatory organisms in stool samples collected between 2-5 days after cardiac surgery with CPB in pediatric patients were previously identified (Salomon et al., 2021). Animal studies with longer periods of observation after intervention are needed to confirm these changes.

The goals for administration of inotropic and vasoactive agents in the CPB/DHCA group were to maintain normal physiological parameters and reduce secondary insults from poor perfusion, elevated central venous pressures or lactic acid production. Although little data is available on the effect of vasoactive agents on the microbiome, there is some data related to administration and drug metabolism from gut bacteria with cardiovascular medications such as statins and anti-hypertensive agents (Wan and Zuo, 2022; Forslund et al., 2021; Yang et al., 2022). Additionally, genetic deletion of vasopressin was associated with microbial shifts (Fields et al., 2018); however, what this means in terms of administering vasopressin intravenously is unknown. There might be alterations to the microbiome from altered intestinal oxygen supply and mucosal oxygen tension (Knotzer et al., 2005), but the relationship of this effect to the microbiome is unknown. Dopamine as a neurotransmitter has been implicated in the gut–brain axis. At this time, data are limited to administration of L-dopa medications related to neurocognitive diseases, such as Parkinson's Disease (Peters, 2019; Gonzalez-Arancibia et al., 2019), and describe the role of the microbiome in altering drug metabolism and availability. It is unknown at this time whether intravenous administration of vasoactive agents alters the gut flora. It is more likely, however, that changes in hemodynamics, hypoxemia and abnormal hemodynamics would have a much larger effect on the intestinal epithelium and microbial shifts than an intravenous vasoactive medication.

This study was performed to determine the feasibility of evaluating similar markers in an animal model of CPB that we currently evaluate in human subjects. Our results are promising, and future examination of specific pathways and shotgun metagenomics can build from this foundation. With 16S metagenomic data, it is not possible to clarify the role of these molecules with changes in the microbiome, especially as interpretation on the species level can be limited compared to shotgun whole-genome sequencing. Whole-genome sequencing would allow appropriate interpretation of genetic pathway signaling and many of these molecules. Based on the data shown in Fig. 6, it is likely that increased levels of inflammatory organisms increasing the production of endotoxins, such as lipopolysaccharides, and decreased levels of SCFA-producing organisms will be potential targets for alterations in the molecules identified in Fig. 7. Future studies to evaluate this in more detail are needed.

Multiple studies have utilized piglet bypass to understand organ dysfunction and cardiac and neurologic injury, as well as techniques for performing CPB and DHCA. No study, to date, has used this method to evaluate relationships with the microbiome and inflammation following CPB. Progress is also being made to grow organoids with specific congenital heart disease malformations, such as hypoplastic left heart syndrome, Tetralogy of Fallot and septal defects (Rufaihah et al., 2021). If introducing these genomic mutations to produce animals with specific cardiac defects is successful, this will improve animal modeling of cardiac defects for the study of various interventions and associated disease processes. As we learn more about the contributions of the microbiome and metabolite profile to the inflammatory process, managing microbial and metabolite aberrations might result in reduced inflammatory signaling and improved clinical outcomes in the post-operative period, especially in animal models that have the same cardiac defects as our human patients. Further studies are needed to develop a causal relationship between microbial and metabolite perturbations and systemic inflammation following CPB, mediated through EBD and intestinal permeability.

## MATERIALS AND METHODS

### Piglet CPB surgery

The animal protocol was approved by the Institutional Animal Care and Use Committee of the University of Colorado [protocol number 107715(02)1D]. This was in accordance with the Guide for the Care and Use of Laboratory Animals and the ARRIVE guidelines (Percie Du Sert et al., 2020). Although the full protocol also included several experimental medical interventions, for the purpose of this secondary study, only those not receiving investigational medications were included. Twelve specific pathogen-free female Yorkshire/Landrace cross pigs, weighing 6-10 kg (8.33±0.82 kg, Oak Hill Genetics, Ewing, IL, USA) were included in this study. Upon arrival to the animal facilities, health status was confirmed by a veterinarian technician. All animals were housed in groups (three to six animals per pen) and given a minimum of 3 days of acclimation. Environmental conditions were 14 h/10 h light:dark cycle, 30-50% humidity and 18-24°C temperature. Water was always readily available and a feeding schedule (Teklad 8753, Envigo, Madison, WI, USA) was based on activity factor of 1.2, for slight growth/gain and maintenance, 100 g fed twice daily.

The animals were divided into two groups, the CPB/DHCA group, which underwent CPB with DHCA, and the control group, which underwent mechanical ventilation. The piglets were made NPO for a total of 10 h prior intervention. The surgical methods have been previously published (Davidson et al., 2019). The piglets were placed on peripheral CPB, which was achieved using isoflurane anesthesia. Once on CPB, the piglets were cooled via the CPB circuit to 22°C, inducing circulatory arrest. DCHA was maintained for 75 min followed by rewarming to 36°C. After rewarming and separation from CPB, the piglets were provided ICU care and hemodynamic monitoring for 4 h and then euthanized. Hemodynamic support included vasoactive and inotropic agents including Milrinone, epinephrine, norepinephrine, dopamine, and vasopressin. Control piglets received no inotropic support during the mechanical ventilation or supportive period. The amount of each of these medications was tabulated to determine the vasoactive inotrope score as a measure for the degree of hemodynamic support the CPB/DHCA group required after separation from CPB. Piglets in the control group were intubated and placed on mechanical ventilation for 7 h with the same ventilator parameters as provided in the CPB/DHCA group.

Parameters for CPB and DHCA included a variable duration of bypass based upon the time to cool and rewarm the piglets. The cooling time ranged from 20-30 min and rewarming time ranged from 30-45 min. Targeted flow of 100 ml/kg/min was achieved with the goal of central venous pressure less than 7 cm H_2_O and mean arterial pressure 40-70 mm Hg. Control piglets received no inotropic support during mechanical ventilation or the supportive period. Targeted mechanical ventilation for both the CPB/DHCA group and control group consisted of pCO_2_ 35-45 mm Hg, peak inspiratory pressure less than 30 (range of 18-22 cm H_2_O in the control group and pre-operative CPB/DHCA group, and 23-28 cm H_2_O in the post-operative CPB/DHCA group), starting ventilation volumes of 13 ml/kg due to the larger pig lung to weight ratio, positive end expiratory pressure of 6 mm Hg, respiratory rate of 16 breaths/min, and fraction of inspired oxygen 1.0. The CPB/DHCA group was exsanguinated into the venous reservoir during DHCA.

### Stool and blood preparation

Stool was obtained just prior to CPB surgery and again at the time of sacrifice by direct removal from the rectum. These samples were frozen at −80°C. Blood samples were collected from piglets after the femoral arterial line was placed, prior to neck cannulation for CPB, and again at the time of sacrifice. These samples were centrifuged, and the serum was separated and stored at −80°C. DNA from the stool samples was extracted using the QIAGEN PowerFecal DNA Extraction kit. Extracted DNA was quantified using the ND-2000C Spectrophotometer (Thermo Fisher Scientific). DNA was then diluted to a standard volume and concentration for 16S rRNA library preparation.

### 16S rRNA library preparation and sequencing

The DNA was quality checked using a Qubit 3.0 fluorometer (Thermo Fisher Scientific) before 16S metagenomic sequencing library preparation following the Illumina MiSeq pair-end protocol. The protocol targeted the variable 16S V3 and V4 regions with the following primer sequences: 16S amplicon PCR forward primer, 5′-TCGTCGGCAGCGTCAGATGTGTATAAGAGACAGCCTACGGGGGCGCAG-3′; 16S amplicon PCR reverse primer, 5′-GTCTCGTGGGCTCGGAGATGTGTATAGTCTCGTGGGCTCGGAGATGTGTATAAGAGACAGGACTACGGGTATCTAATCC-3′. After libraries were quantified and normalized, a 4 nM pool of all samples was denatured and diluted to 8 pM. This pool was loaded to the Illumina MiSeq for a 300 bp paired-end run using the MiSeq v3 600 cycle kit.

### Sequencing bioinformatics and microbiome analysis

Sequences were demultiplexed using Illumina software (MiSeq Control Software version 2.6) according to the manufacturer's guidelines. After the demultiplexing step, the bioinformatics analyses were performed following the Bioconductor workflow for microbiome data analysis (Callahan et al., 2016b) using R software (version 4.0).

For denoising, the R package DADA2 (version 1.18.0) (Callahan et al., 2016a) was used with the following conditions: the forward reads were truncated at position 280 and their first 17 nucleotides were trimmed, whereas the reverse ones were truncated at the position 250 and their first 21 nucleotides were trimmed, to discard positions for which nucleotide median quality was Q25 or below. High-quality sequencing reads were clustered to infer amplicon sequence variants (ASVs), and a final table of ASV counts per sample was generated after removing chimeras. In addition, a naïve Bayes taxonomy classifier (Wang et al., 2007) was used to classify each ASV against the SILVA 138.1 reference database to construct the taxonomy table, and MAFFT (version 7.407) (Katoh et al., 2002) and FASTTREE (version 2.1.11) (Price et al., 2009) programs were used to construct a phylogenetic tree.

Taxa abundances were normalized with the total sum scaling normalization method dividing each ASV count by the total library size to yield their relative proportion of counts for each sample. α-diversity was studied with the R packages phyloseq (version 1.34.0) (McMurdie and Holmes, 2013) and picante (version 1.8.2) (Kembel et al., 2010). Principal coordinates analysis (PCoA) via a UniFrac distance matrix was used to evaluate β-diversity and to plot patterns of microbiome community diversity.

Permutational multivariate analysis of variance (PERMANOVA) of the distance matrices was performed with 999 permutations as implemented in the R package vegan (version 2.5-7) (https://CRAN.R-project.org/package=vegan) to reveal statistical significance. Linear discriminant analysis (LDA) effect size (LEfSE) analysis (version 1.1.2) (Segata et al., 2011) was performed. Differential abundance analyses were performed using R packages AMCOM-BC (version 1.0.5) (Lin and Peddada, 2020) and corncob (version 0.2.0) (Martin et al., 2020) to reveal statistically significantly changed taxa. For taxa differential abundance analysis, Benjamini–Hochberg procedure was applied to correct for multiple hypothesis testing.

Canonical correlation analyses were performed using the R package mixOmics (version 6.14.1) (Rohart et al., 2017) to explore correlation between microbiome and plasma biomarkers and/or stool metabolites. Mediation analysis was performed using the Modima method by Hamidi et al. (2019) to evaluate the mediation effect of the microbiome using CPB surgery as exposure, and plasma and stool biomarkers as outcomes.

### ELISA and immunoassay multiplex

Claudin-2, claudin-3 and FABP2 in piglet arterial serum samples from control and CPB/DHCA groups were analyzed via enzyme-linked immunosorbent assay (ELISA) (MyBioSource) in a 96-well plate prepared according to the manufacturer's instruction. Captured antibody-precoated plates were incubated with standards and samples for 90 min at 37°C, biotin-labeled antibodies were transferred and incubated for 60 min at 37°C, and HRP-conjugated streptavidin was transferred and incubated for 30 min at 37°C, followed by light-protected incubation with 3,3′,5,5′-tetramethylbenzidine (TMB) for 20 min at 37°C. Samples were normalized with a standard volume and ran in duplicate. Stop solution was added and the absorbance was measured immediately at 450 nm using a microplate reader (Molecular Devices, SpectraMax M3).

Arterial serum interleukin-1β (IL-1β), interleukin-6 (IL-6), and tumor necrosis factor-α (TNF-α) were measured by using commercially available multiplex magnetic bead-based immunoassay kits (R&D Systems). Briefly, 50 μl of standard or samples were added to a 96-well plate, followed by addition of 50 μl of diluted microparticle cocktail and incubation for 2 h at room temperature. The diluted biotin-antibody cocktail of 50 μl was added to each well and incubated for 1 h at room temperature. Then, 50 μl of diluted streptavidin-PE was transferred and incubated for 30 min at room temperature. Lastly, 100 μl of wash buffer was added to each well immediately using Luminex 200 (Thermo Fisher Scientific).

### Metabolomics

Metabolomic profiling for eicosanoids and short-chain fatty acids was performed when there was sufficient remaining stool sample. Eicosanoids were quantitated using ultra-performance liquid chromatography-tandem mass spectrometry (UPLC-MS/MS) as previously described (Salomon et al., 2021) and similarly to other metabolomic approaches (Weckerle et al., 2022). Analysis of the eicosanoids and SCFAs involved converting the raw data to concentration data (ng/g for stool weight) using the Lab Solution software (version 5.99) (Shimadzu Scientific, Columbia, MD, USA). Briefly, samples were standardized for dehydration and mass prior to analysis. Separation and quantitation were performed using a Nexera UPLC system coupled with a Shimadzu 8060NX mass spectrometer (Shimadzu Scientific Instruments, Columbia, MD, USA). Unlabeled eicosanoids and isotope-labeled internal standards were obtained from Cayman Chemicals (Ann Arbor, MI, USA). Eicosanoids were measured in arterial serum for samples in both groups and similar protocols were followed for standardization of mass and dehydration as performed on the stool samples. All eicosanoids were detected in negative ionization mode. Metabolomic eicosanoid data analysis was performed using MetaboAnalyst 5.0 online platform (https://www.metaboanalyst.ca/).

For SCFAs, a validated UPLC-MS/MS was also used to evaluate samples (Samuelson et al., 2022). In total, eight SCFAs were quantitated. The SCFAs were extracted from the stool and prepared with quality control and calibration standards, by a simultaneous extraction/derivatization pre-treatment procedure. Stool samples were weighed, homogenized, vortexed, further diluted with water, and then centrifuged (3210 ***g***). The resulting supernatant was derivatized with 3-nitrophenylhydrazine,N-(3-dimethylaminopropyl)-N-ethylcarbodimide hydrochloride (TCI Chemicals) and pyridine (Thermo Fisher Scientific) prior to analysis.

### Statistical analysis

Animal clinical characteristics and laboratory variables were analyzed by study group. Data were tested for normality using the Shapiro–Wilk method. For normally distributed variables, we report means with s.e.m. For non-normally distributed variables, logarithmic conversion was performed to normalize the data set. For EBD markers, SCFAs and cytokines, the CPB/DHCA group was compared to the control group with two-way ANOVA for normally distributed variables and mixed-effects analysis if any data time points were unavailable (i.e. not enough sample to run all the experiments for a given animal) using GraphPad Prism (version 9.3.1, GraphPad Software, San Diego, CA, USA). Multiple comparison analysis using Holm–Sidak's correction was performed. A *P*-value of 0.05 was used as the cutoff for statistical significance for all data sets, including microbiome analysis, EBD markers, cytokines, SCFAs and eicosanoids.

## Supplementary Material

10.1242/dmm.049742_sup1 Supplementary information
