# Supplementary material for: Piglet cardiopulmonary bypass induces intestinal dysbiosis and barrier dysfunction associated with systemic inflammation
Source: Dis Model Mech. 2023 Jan 12;16(5):dmm049742. doi: 10.1242/dmm.049742 (PMC9844230; doi:10.1242/dmm.049742)
Supplement: Supplementary information [file dmm-16-049742-s1.pdf]

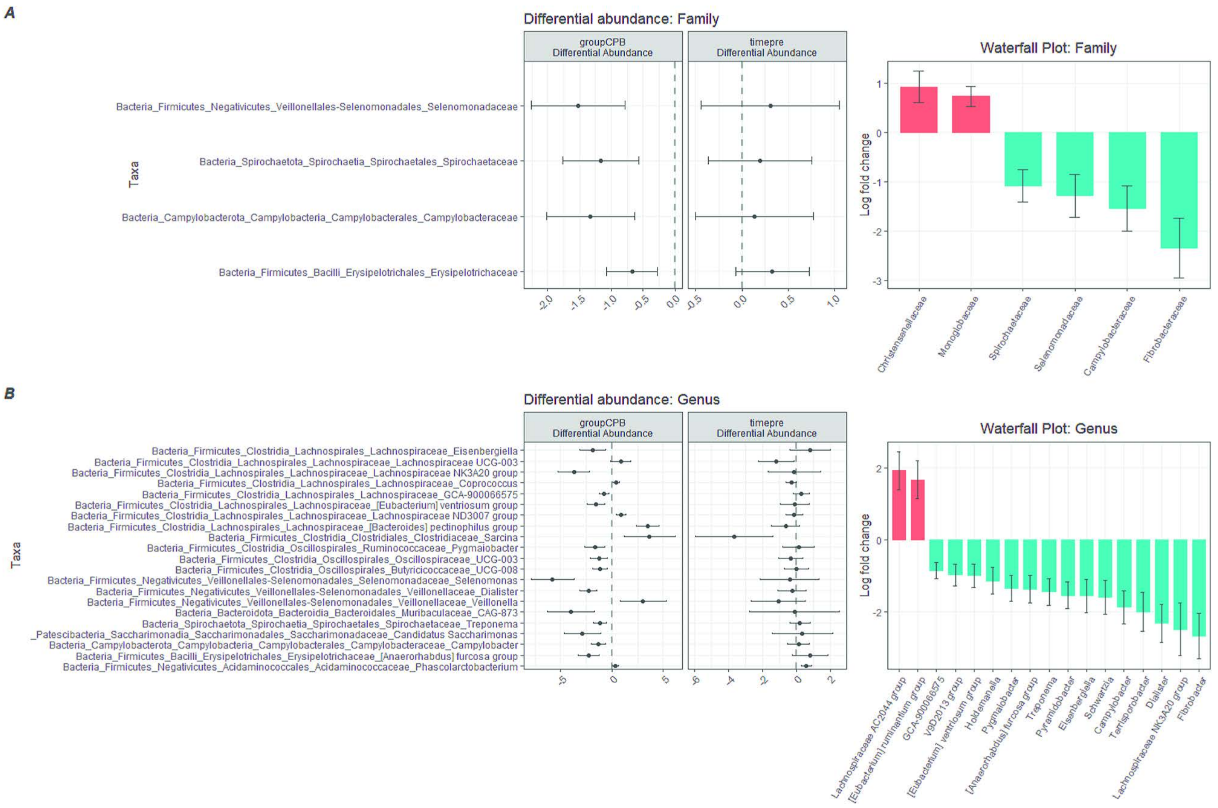

Fig. S1. Differential Abundance in the CPB/DHCA Group at the Family and Genus levels. Panel A depicts taxonomic family rank changes in organism abundance of the CPB/DHCA group showing waterfall plot (top) and the corncob plot (bottom). Panel B depicts taxonomic genus rank changes in organism abundance of the CPB/DHCA group with waterfall plot (top) and corncob plot (bottom). Both waterfall plot and corncob plot show LOG fold changes of organisms that are different between the two groups.

Disease Models & Mechanisms • Supplementary information

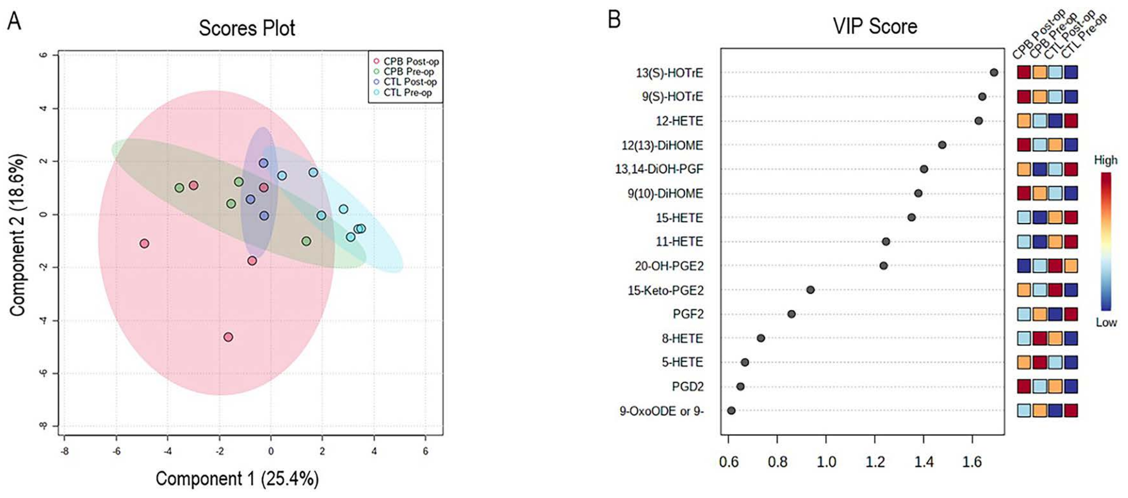

Fig. S2. Partial least squares discriminate analysis and VIP scoring for Stool Eicosanoids in the CPB/DHCA Group and Controls. Panel A shows a two dimensional score plot with differences between the two groups and time points for each group. Panel B shows a VIP score for different eicosanoids in each group based upon the results from the PLS-DA model.

CPB, Cardiopulmonary bypass; CTL, Control; VIP, Variable importance in PLS-DA model.

Disease Models & Mechanisms • Supplementary information

Table S1. Hemodynamic Support and Vasoactive Inotrope Score Following CPB

Piglets	Vasoactive Inotrope Score	Milrinone mcg/kg/min	Dopamine mcg/kg/min	Epinephrine mcg/kg/min	Vasopressin mcg/kg/min	Event
**Piglet #1**						
Hour 0	12	0.5	2	0.05	0	Off bypass
Hour 1	20	0.5	2	0.08	0.0005	
Hour 2	23	0.3	2	0.08	0.001	
Hour 3	23	0.3	2	0.08	0.001	
Hour 4	23	0.3	2	0.08	0.001	Pre-euthanasia
**Piglet #2**						
Hour 0	19	0.5	5	0.09	0	Off bypass
Hour 1	19	0.5	5	0.09	0	
Hour 2	19	0.5	5	0.09	0	
Hour 3	19	0.5	5	0.09	0	
Hour 4	19	0.5	5	0.09	0	Pre-euthanasia
**Piglet #3**						
Hour 0	15	0.5	5	0.05	0	Off bypass
Hour 1	15	0.5	5	0.05	0	
Hour 2	15	0.5	5	0.05	0	
Hour 3	16	0.3	5	0.08	0	
Hour 4	16	0.3	5	0.08	0	Pre-euthanasia
**Piglet #4**						
Hour 0	12.7	0.5	2	0.05	0	Off bypass
Hour 1	10.7	0.3	2	0.05	0	
Hour 2	10.7	0.3	2	0.05	0	
Hour 3	10.7	0.3	2	0.05	0	
Hour 4	10.7	0.3	2	0.05	0	Pre-euthanasia
**Piglet #5**						
Hour 0	12	0.5	2	0.05	0	Off bypass
Hour 1	12	0.5	2	0.05	0	
Hour 2	10	0.3	2	0.05	0	
Hour 3	7	0	2	0.05	0	
Hour 4	7	0	2	0.05	0	Pre-euthanasia

CPB, Cardiopulmonary bypass; mcg, microgram; kg, kilogram; min, minute

Disease Models & Mechanisms • Supplementary information
